# Surgical implantation of human adipose derived stem cells attenuates experimentally induced hepatic fibrosis in rats

**DOI:** 10.1186/s10020-022-00566-6

**Published:** 2022-11-29

**Authors:** Masateru Nomura, Joseph George, Chieko Hashizume, Takashi Saito, Yoshimichi Ueda, Yasuhito Ishigaki, Mutsumi Tsuchishima, Mikihiro Tsutsumi

**Affiliations:** 1 Department of Hepatology, Kanazawa Medical University, Uchinada, Ishikawa 920-0293 Japan; 2 Center for Regenerative Medicine, Kanazawa Medical University Hospital, Uchinada, Ishikawa 920-0293 Japan; 3 Department of Pathology II, Kanazawa Medical University, Uchinada, Ishikawa 920-0293 Japan; 4 Medical Research Institute, Kanazawa Medical University, Uchinada, Ishikawa 920-0293 Japan

**Keywords:** Adipose derived stem cells, hADSCs, Mesenchymal stem cells, MSCs, Hepatic fibrosis, Liver cirrhosis, Carbon tetrachloride

## Abstract

**Background:**

Mesenchymal stem cells (MSCs) are multipotent stromal cells and could exert hepatoprotective effects against acute liver injury, steatohepatitis, and fibrogenesis. Here, we evaluated the effects of human adipose derived stem cells (hADSCs) to attenuate experimentally induced hepatic fibrosis and early cirrhosis in rats.

**Methods:**

Hepatic fibrosis was induced by intraperitoneal injections of CCl_4_ (0.1 ml/100 g body weight) twice a week for 8 weeks. hADSCs were isolated and cultured on polyethylene discs coated with hydroxyapatite and 2 cm diameter disc was surgically implanted on the right lateral lobe of the liver. Discs implanted without hADSCs served as control. The animals were injected again with CCl_4_ once a week for another 8 weeks. All the animals were sacrificed at the end of 16th week.

**Results:**

Serial administrations of CCl_4_ resulted in well developed fibrosis and early cirrhosis at 8th week which maintained until the 16th week. Animals treated with hADSC discs depicted over 50% decrease of collagen with significant increase in serum albumin and total protein levels. Immunohistochemical staining for TGF-β1, α-smooth muscle actin, and collagen type I and type III demonstrated marked decrease compared to the animals without hADSC treatment.

**Conclusions:**

Treatment with hADSCs improved liver functions, markedly reduced hepatic fibrosis and early cirrhosis. Various pleiotropic and paracrine factors secreted from the hADSCs seem to serve as reparative functions in the attenuation of liver cirrhosis. The data demonstrated that treatment with hADSCs can be successfully used as a potent therapeutic method to prevent progression of hepatic fibrosis and related adverse events.

## Introduction

Hepatic fibrosis and cirrhosis are conditions that are defined as excessive synthesis and deposition of connective tissue components, especially fibril forming collagens in the extracellular matrix of the liver (Acharya et al. 2021; George and Chandrakasan 1996). It is a continuous wound healing process in response to a persistent liver injury mediated by oxidative stress and a variety of inflammatory cytokines and growth factors (George et al. 2019a, 2001). The condition has a variety of clinical manifestations and complications including ascites, portal hypertension, hepatic encephalopathy, which can be life-threatening (D'Amico et al. 2006; Nusrat et al. 2014). Regardless of the cause, the development of hepatic fibrosis and liver cirrhosis consists of accumulation of thick collagen fibers in the hepatic parenchyma that leads to distortion of lobular architecture and formation of regenerative nodules (George et al. 2020, 2017). These processes results in decrease of hepatic mass, alteration of blood flow, esophageal varices, and could ultimately lead to hepatocellular carcinoma. Prognosis of liver cirrhosis is poor, especially when ascites is present with impaired liver function and high Child–Pugh score (Yatsuhashi et al. 2021; Matsue et al. 2015). The only effective and available treatment for end-stage liver cirrhosis is liver transplantation (Esquivel 2010). Due to scarcity of donors, complications associated with transplantation, and organ rejection, it is important to develop alternative treatment modalities to improve liver function and quality of patient's life.

Mesenchymal stem cells (MSCs) are multipotent adult stem cells and can be isolated from various sources such as bone marrow, adipose tissue, and umbilical cord blood. They can differentiate into multiple tissues including bone, cartilage, muscle, adipose, and connective tissue (George et al. 2006). MSCs have a remarkable capacity for extensive in vitro expansion, which allow them to rapidly reach the desired number for in-vivo therapy (Ajami et al. 2019). The beneficial impact of MSCs could be attributed to their pleiotropic mode of action comprising immuno-modulatory, anti-inflammatory, anti-oxidative, antifibrotic, anti-apoptotic, and pro-regenerative features, that are involved in tissue restoration after injury or impairment (Glenn and Whartenby 2014; Minteer et al. 2015). The MSCs sense the microenvironment of the injury site and secrete various paracrine factors that trigger several regenerative functions, in response to environmental cues to enhance repair and restoration of the damaged tissue (Park et al. 2018; Frese et al. 2016).

Several studies have demonstrated that MSCs could markedly attenuate or prevent ischemic liver injury, acute liver failure, steatohepatitis, and hepatic fibrosis (Christ et al. 2015; Winkler et al. 2014; Zagoura et al. 2012). In most of the studies, MSCs were injected intravenously through tail vein in rodent models of experimentally induced liver injury or chronic liver diseases. In such instances, MSCs are distributed throughout the body and various organs after the intravenous injections and not localized in the liver. Therefore, it is important to investigate the therapeutic and regenerative effect of MSCs after implantation in the injured or diseased liver tissue. In the current study, we cultured human adipose tissue derived stem cells (hADSCs) on a polyethylene disc coated with hydroxyapatite and then implanted the disc with the cells on the surface of right lateral lobe of the liver tissue of rats treated with CCl_4_ to induce hepatic fibrosis. We evaluated whether hADSCs attenuate CCl_4_ induced liver injury and fibrosis and also whether the hADSCs differentiate into hepatic progenitor cells.

## Materials and methods

### Isolation, culture, and expansion of human adipose tissue derived stem cells

Human ADSCs were isolated from abdominal adipose tissue collected during liposuction for cosmetic purposes. The study protocol was reviewed and approved by the Ethics and Clinical Investigation Committee of Kanazawa Medical University, Ishikawa, Japan (#G129) and has been conducted according to the principles outlined in the Declaration of Helsinki (revised 2013). Written consent was obtained from each patient undergone liposuction from abdominal subcutaneous adipose tissue during cosmetic procedures. About 30 ml of lipoaspirate was collected and processed with Lipogems device (Cat# LG PK 240, Lipogems International, Milan, Italy) and obtained 10 ml of final Lipogems product as per the manufacturer's protocol (Bianchi et al. 2013).

**Fig. 1:**
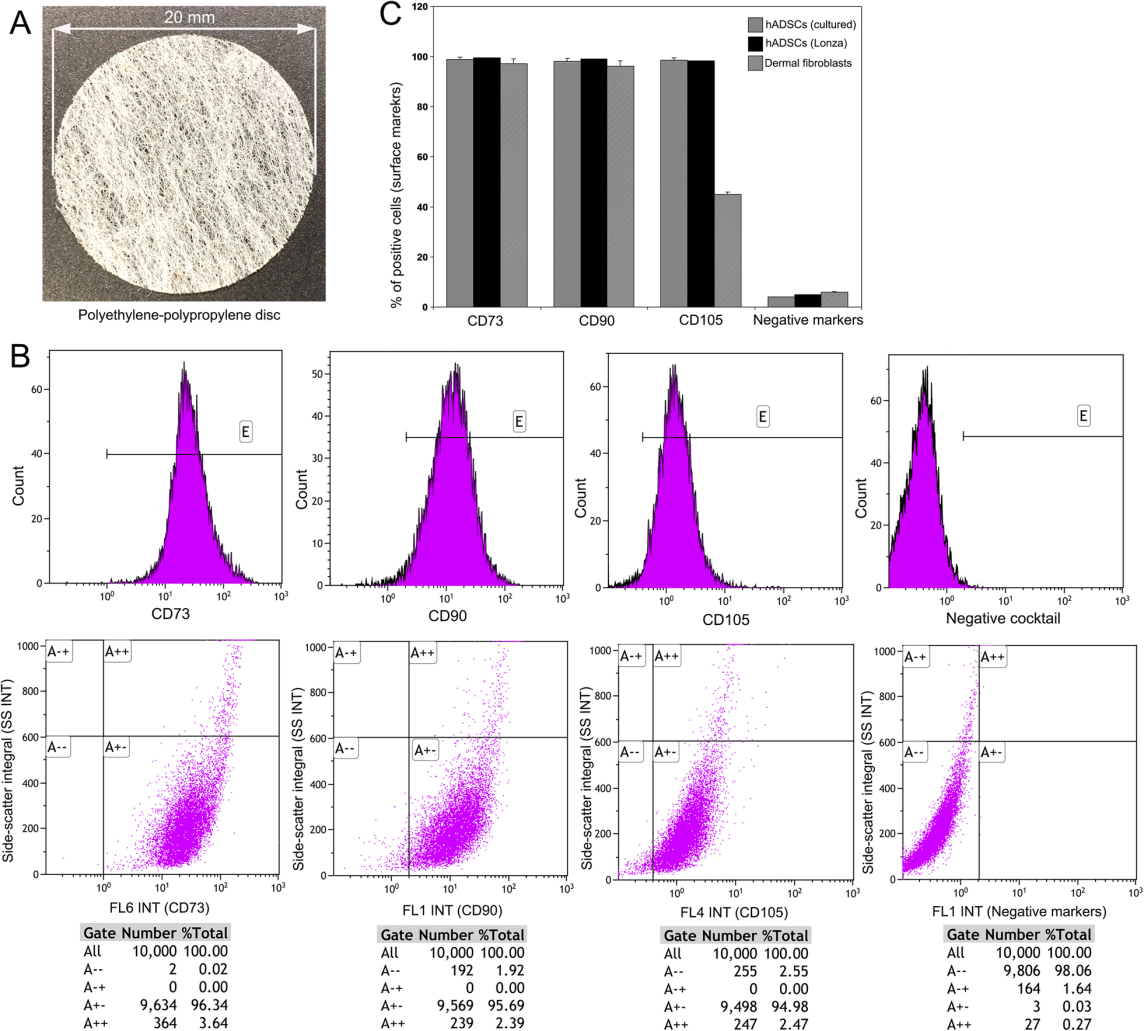
**A** Photographic image of non-woven fabric polyethylene-polypropylene (PE-PP) disc coated with hydroxyapatite used for isolation and culture of human adipose tissue derived stem cells (hADSCs) without enzymatic digestion. **B** Representative histogram and dot plot images of the flow cytometric analysis of isolated and cultured hADSCs (2nd passage) for the expression of CD73, CD90, and CD105 and a cocktail of negative markers (CD34, CD11b, CD19, CD45, and HLA-DR) after harvested using Accutase cell detachment solution and treatment with respective anti-human mAbs conjugated with various fluorescent tags. The gated cells in A +—column indicates they are positive for the respective surface marker and the cells in A– column indicates that the negative markers are not expressed in hADSCs. Practically no cells are present in A +—column with the expression of negative markers. **C** Graphical presentation of the flow cytometry data for the expression of surface markers in cultured hADSCs, human ADSCs from Lonza, and human dermal fibroblast cells. The data are mean ± S.D. of 5 independent experiments

The adipose stem cells present in the Lipogems were isolated and purified using a kit (Cat# BMK-R001, Bio Future Technologies, Tokyo, Japan), which allows isolation of adipose stem cells without enzymatic digestion. The technique utilizes the property of adipose tissue stem cells to trap inside a three-dimensional fibrous network coated with hydroxyapatite, which allows the stem cells to proliferate fast (Fig. 1A). Inside a cell culture cabinet, a 2 cm diameter non-woven fabric polyethylene-polypropylene (PE-PP) disc coated with hydroxyapatite was placed in a cell strainer (both provided in the kit, Cat# BMK-R001) and poured 1 ml of the above Lipogem suspension on top of the disc. The disc was rinsed twice using sterile PBS pouring gently on top of the disc with a pipette. Then with tweezers, the disc with adipose stem cells was transferred to an uncoated 60 mm cell culture dish containing KBM ADSC-1 medium (Cat# 16030020, Kohjin Bio, Saitama, Japan). It was incubated at 37 °C in a humidified atmosphere with 5% CO_2_ on air without changing the medium for one week. The medium was changed after a week and examined the disc under a phase contrast microscope to confirm the presence of fibroblast-like adipose stem cells proliferating on the fibrous network of the disc. After 2 weeks, when hADSCs were proliferated around 20% of the surface area of the disc, the culture was washed twice with serum free media. Then the adherent cells on the disc were treated with trypsin–EDTA solution (Cat# 204-16935, FUJIFILM Wako Chemicals, Tokyo, Japan) and harvested. This process yielded about 1 million cells per disc. The cells were washed with serum free media and cultured in KBM ADSC-1 medium in a 10 ml culture dish until the culture attained 80% confluence. It took about 5 days and produced around 10 million cells per dish. The cells were harvested using trypsin–EDTA solution, and tested for mycoplasma contamination using mycoplasma detection kit (Cat# LT07-118, Lonza, Walkersville, MD, USA). A portion of the cells were analyzed on a FACS machine for various markers of stem cell and human origin. The remaining cells were frozen as 1 million cells/vial and stored in liquid nitrogen.

### Flow cytometry

Cultured hADSCs (2nd passage), human ADSCs from Lonza (Cat# PT-5006, Walkersville, MD, USA), and human dermal fibroblast cells (HDFCs) from Kurabo (Cat# KF-4109, Chuo-ku, Osaka, Japan) were treated with Accutase (Cat# 561527, BD Biosciences, San Jose, CA, USA). After centrifugation, the cell pellet was suspended in stain buffer (Cat# 554656, BD Biosciences) to a concentration of 0.5–1.0 × 10^7^ cell/ml. After blocking with human FcR blocking reagent (Cat# 130-059-901, Miltenyi Biotec, Bergisch Gladbach, Germany), the cells were treated with various anti-human mAbs (raised in mouse) conjugated either with FITC, phycoerythrin (PE), PerCP-Cy5.5, or allophycocyanin (APC) for 30 min at 4 °C. The following mAbs are used: anti-CD73-APC, anti-CD90-FITC, anti-CD105-PerCP-Cy5.5, and negative cocktail (anti-CD34-PE, anti-CD45-PE, anti-CD11b-PE, anti-CD19-PE, and anti-human-leukocyte antigen (HLA) -DR-PE) (Cat# 562245, Human MSC analysis kit, BD Biosciences, San Jose, CA). Cells were analyzed on a Gallios flow cytometer with Kaluza software (Beckman Coulter, Brea, CA, USA). Positive and negative cells were counted and compared with the signal of corresponding immunoglobulin isotypes.

### Differentiation of hADSCs into adipocytes and osteocytes

In order to evaluate the differentiation potency of purified hADSCs into adipocytes and osteocytes, we cultured 2nd passage of hADSCs in human mesenchymal stem cell adipogenic differentiation medium BulletKit (Cat# PT-3004, Lonza, Walkersville, MD, USA) or osteogenic differentiation medium BulletKit (Cat# PT-3002, Lonza). After 2–3 weeks of culture, adipogenic differentiation was identified using AdipoRed (Cat# PT-7009, Lonza, Walkersville, MD) staining for the lipid droplets and osteogenic differentiation was characterized using Alizarin Red (Cat# ARD-A1, PG Research, Kodaira, Tokyo, Japan) staining for the mineralized matrix. As control cells, human ADSCs were purchased from Lonza (Cat# PT-5006) and human dermal fibroblasts (Cat# KF-4109) were obtained from Kurabo, Chuo-ku, Osaka, Japan.

### Culture of hADSCs on polyethylene disc for surgical implantation

A vial of 2nd passage of purified and characterized hADSCs stored in liquid nitrogen was removed and thawed at 37 °C water bath. A 2 cm diameter polyethylene-polypropylene (PE-PP) disc coated with hydroxyapatite (Cat# BMK-R001, Bio Future Technologies, Tokyo, Japan) was inserted into a 2 ml sterile eppendorf tube with the help of tweezers. The thawed hADSCs were gently pipetted into the eppendorf tube containing the disc and kept in a CO_2_ incubator at 37 °C with intermittent gentle shaking for 3 h. This process allowed adipose stem cells to trap inside the three-dimensional fibrous network and adhere to the fibers. The disc attached with the hADSCs was transferred to a cell culture dish with KBM ADSC-1 medium (Cat# 16030020, Kohjin Bio, Saitama, Japan) and incubated at 37 °C in a humidified atmosphere with 5% CO_2_ on air for 5–7 days. When the disc attained around 80% confluence, it was washed in serum free media followed in sterile PBS and used for surgical implantation on the dorsal surface of rat liver.

### Animals and experimental procedures

Male albino rats of Wistar strain at the age of around 2 months and weighing between 200–230 g were used for the experiments. The animals were procured from Japan SLC Corporation (SLC, Hamamatsu, Shizuoka, Japan). They were housed in temperature and humidity controlled animal house under 12-h light/12-h dark cycles with commercial rat feed pellets and water available ad libitum. All the animal experiments were carried out following the *Guide for the Care and Use of Laboratory Animals* published by the US National Institutes of Health (NIH Publication No. 86-23, revised 1996). The animal experimental protocol was also approved by the Animal Care and Research Committee of Kanazawa Medical University on the ethics for the care and use of laboratory animals (#2017-89).

Fifteen rats were injected with CCl_4_ (Wako, Tokyo, Japan) at a dose of 0.1 ml/100 g body weight (diluted to 50% in olive oil) intraperitoneally twice a week for 8 weeks. Another 5 rats received similar injections without CCl_4_ and served as control. At the end of 8 weeks, 5 rats from the CCl_4_ group were euthanized to evaluate the development of hepatic fibrosis and early cirrhosis. The remaining 10 rats were randomly divided into two groups of 5 rats each. Under isoflurane anesthesia, the abdomen was shaved and disinfected with 70% ethanol. An upper abdominal incision was made and a 2 cm diameter disc with more than 80% confluence of hADSCs (hADSC group) or a disc without hADSCs (hADSC control group) was implanted on the surface of right lateral lobe of the liver and the laparotomy was closed with suture. All the animals in both hADSC group and hADSC control group were injected with the same dose of CCl_4_ once a week for another 8 weeks to maintain liver fibrosis. The control group received similar dosage of olive oil. At the end of 16th week from the beginning of exposure, all the animals were anaesthetized with isoflurane and blood was collected from right jugular vein. The liver was rapidly removed and 3 mm thick tissue was cut from both right and left lateral lobes and instantly fixed in 10% phosphate-buffered formalin for histopathological studies.

### Measurement of AST, ALT, total protein, and albumin in serum

Blood was allowed to clot at 37 °C and serum was separated in the conventional method. Aspartate aminotransferase (AST), alanine aminotransferase (ALT), total protein, and albumin levels in the serum were measured using an auto-analyzer for animal samples. AST and ALT values are presented as International Units per liter (IU/liter) of serum. Total protein and albumin were depicted as g/100 ml serum.

### Histopathological evaluation of liver tissue

The formalin-fixed liver tissues were processed in an automatic tissue processor optimized for liver tissue, embedded in paraffin blocks, and cut into sections of 5-μm thickness. In hADSC group, the liver tissue was processed from right lateral lobe with disc and left lateral lobe without disc. The liver sections were stained with Azan trichrome stain for connective tissue as per the standard protocol. The stained sections were examined using an Olympus BX53 microscope (Olympus Corporation, Tokyo, Japan) attached with Olympus DP71 digital camera (Olympus Corporation, Tokyo, Japan) and photographed.

### Immunohistochemical staining for TGF-β1, α-SMA, collagen type I, and type III

Immunohistochemical staining was performed for transforming growth factor-β1 (TGF-β1), α-smooth muscle actin (α-SMA), collagen type I, and collagen type III on paraffin liver sections to assess the degree of hepatic fibrosis and cirrhosis. The liver sections were deparaffinized with xylene and alcohol and hydrated to water. Immunohistochemistry was performed employing a broad-spectrum histostain kit (Invitrogen, Carlsbad, CA, USA). After blocking, the liver sections were treated with primary antibodies (Abcam, Chuo-ku, Tokyo, Japan) against TGF-β (Cat# ab92486), α-SMA (Cat# ab5694), type I collagen (Cat# ab34710), and type III collagen (Cat# ab23445) separately and incubated in a moisturized slide chamber (Evergreen Scientific, Los Angeles, CA, USA) at 4 °C overnight. The sections were then washed 3–5 times in cold phosphate-buffered saline and incubated with broad-spectrum biotinylated secondary antibody for 2 h at room temperature. The slides were washed again and treated with streptavidin-peroxidase conjugate and incubated for another 1 h at room temperature. The final stain was developed by using 3% 3-amino-9-ethylcarbazole (AEC) in N, N-dimethylformamide. The stained sections were washed and counterstained with Mayer’s hematoxylin for 2 min and mounted by using aqueous-based mounting medium. The slides were allowed to dry and examined under a microscope (Olympus BX53, Tokyo, Japan) attached with a digital camera (Olympus DP71) and photographed. The staining intensity in 10 randomly selected microscopic fields was quantified by using Image-Pro Discovery software (Media Cybernetics, Silver Spring, MD, USA).

### Staining for hADSC markers Lamin B1 and CD73

In order to confirm the presence of characteristic marker proteins of hADSCs at 8 weeks after implantation, immunohistochemical staining was performed for human Lamin B1 as a marker for human origin employing Lamin B1 antibody (Cat# HS-404013, Synaptic Systems, Goettingen, Germany) and CD73 as a marker for stem cell using CD73 antibody (Cat# 12231-1-AP, Proteintech, Rosemont, IL, USA). The staining protocol was essentially same as described above, except that 3,3′-diaminobenzidine (DAB) was used for the final stain development.

### Statistical analysis

Arithmetic mean and standard deviation (SD) were calculated for all the data and presented as mean ± SD. All the data were analyzed and compared either by using analysis of variance or Student's *t* test. A value of P < 0.05 was considered statistically significant.

## Results

### Culture and characterization of hADSCs

A photographic image of the polyethylene-polypropylene (PE-PP) disc coated with hydroxyapatite used for isolation and culture of hADSCs without enzymatic digestion is depicted in Fig. 1A. The disc was like a thin fabric around the thickness of 0.1 mm. The flow cytometric analysis of isolated and cultured hADSCs (2nd passage) for the expression of CD73, CD90, and CD105 and a cocktail of negative markers (CD34, CD11b, CD19, CD45, and HLA-DR) are presented in Fig. 1B. Flow cytometry demonstrated that more than 95% of cells express CD73, CD90, CD105 and less than 5% cells express CD34, CD11b, CD19, CD45, and HLA-DR. There was no difference in the rate of expression between cultured hADSCs and human ADSC procured from Lonza. Figure 1C shows graphical presentation of the flow cytometry data for the expression of surface markers in cultured hADSCs, human ADSCs from Lonza, and human dermal fibroblast cells.

### Differentiation of hADSCs into adipocytes and osteocytes

**Fig. 2:**
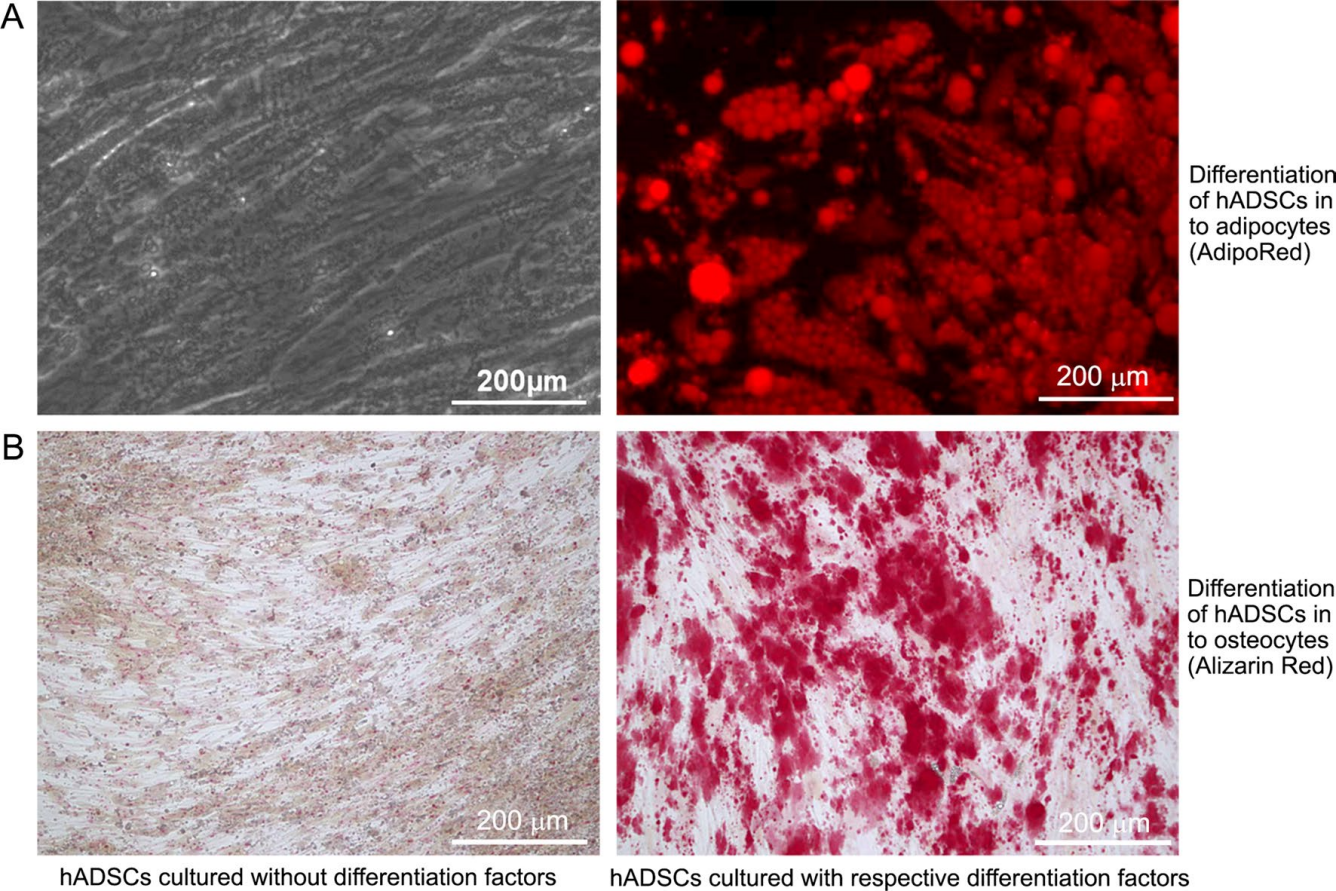
Differentiation of hADSCs into adipocytes and osteocytes. The hADSCs were cultured with respective differentiation medium for 17 days. **A** hADSCs cultured with and without adipogneic differentiation factors and stained with AdipoRed. **B** hADSCs cultured with and without osteogenic differentiation factors and stained with Alizarin Red. Original magnification, × 200

Figure 2 depicts the differentiation of purified hADSCs cultured in respective medium into adipocytes and osteocytes. The adipocyte differentiation was demonstrated with AdipoRed, which stains lipid droplets as red (Fig. 2A). The differentiated osteocytes were treated with Alizarin Red which stains the mineralized matrix (Fig. 2B). This proved that the hADSCs employed in the current study can be differentiated into various mesenchymal cells when treated with appropriate differentiation medium and growth factors.

### Serum levels of AST, ALT, total protein, and albumin in CCl_4_ induced liver cirrhosis and the effect of treatment with hADSCs

**Fig. 3:**
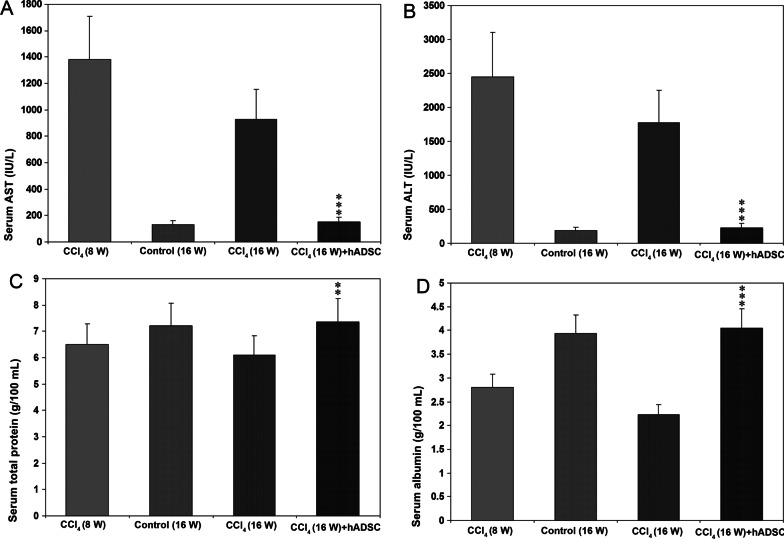
Aspartate aminotransferase (AST), alanine aminotransferase (ALT), total protein (TP), and albumin levels in the serum following serial administrations of CCl_4_ and after treatment with hADSC disc. **A** AST levels **B** ALT levels **C** Serum total protein **D** Serum albumin. Treatment with hADSC disc resulted in restoration of normal levels of AST, ALT, TP, and albumin in the serum. The data are mean ± S.D. of 5 rats in each group. **P < 0.01 and ***P < 0.001 CCl_4_ (16 W) treated group versus CCl_4_ (16 W) + hADSC group

Serum AST levels were highly elevated in rats treated with CCl_4_ for 8 weeks (Fig. 3A), which were returned to almost normal levels after 8 weeks of hADSCs treatment. There was no significant difference in serum AST levels between control and hADSC groups. Serum ALT levels depicted similar pattern as in the case of serum AST levels both in treated and control groups (Fig. 3B).

The decreased total protein levels (*P* < 0.01) were restored to normal levels after treatment with hADSC disc (Fig. 3C). The serum albumin levels were significantly (*P* < 0.001) decreased both at 8 and 16 weeks after CCl_4_ treatment compared to olive oil treated controls (Fig. 3D). Treatment with hADSC disc for 8 weeks restored normal albumin level in animals with liver cirrhosis (Fig. 3D).

### Treatment with hADSCs prevented CCl_4_ induced liver cirrhosis in rats

**Fig. 4:**
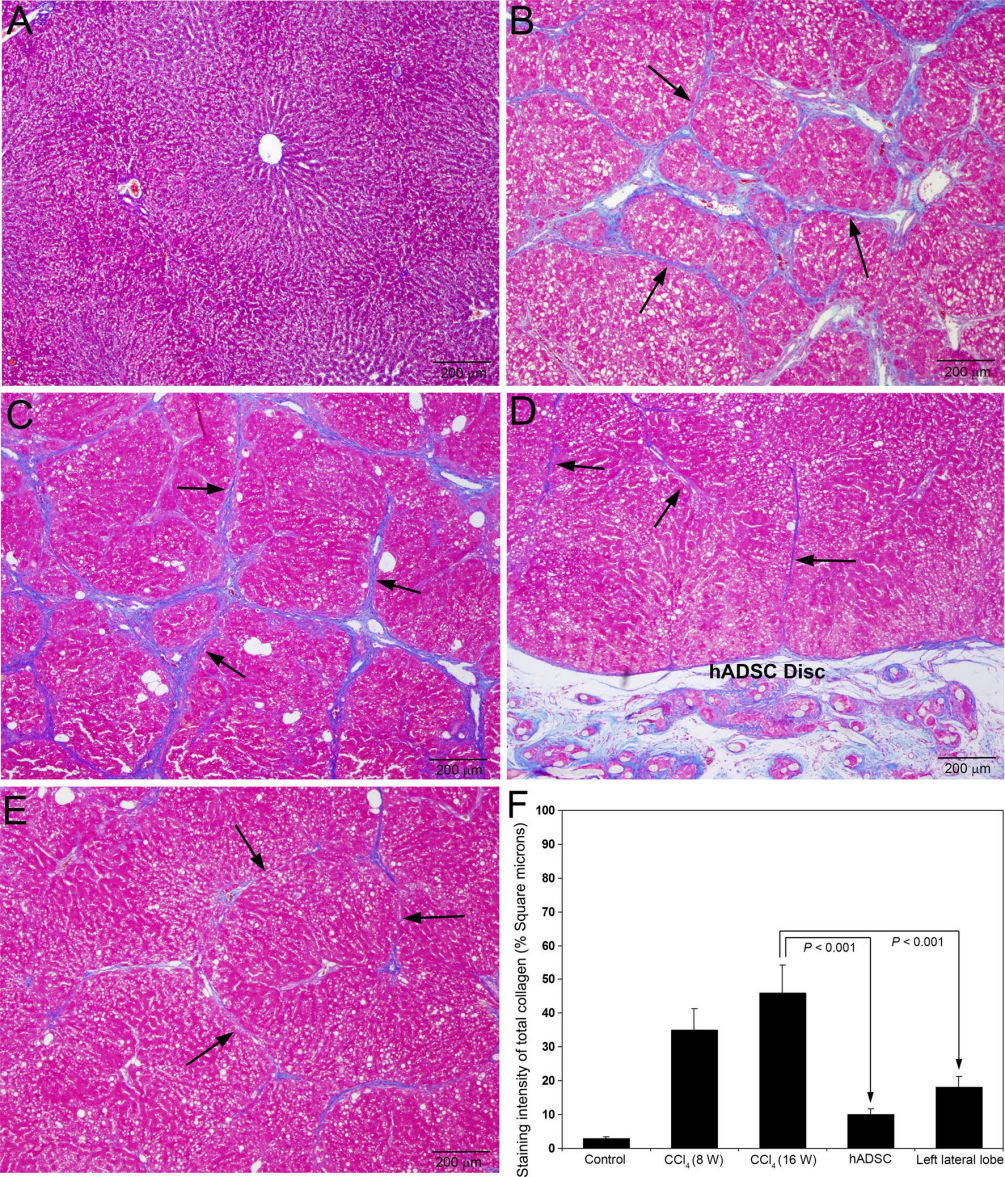
Azan trichrome staining for collagen in rat liver sections following serial administrations of CCl_4_ and after treatment with hADSC disc. **A** Staining for collagen was absent in the control livers treated with olive oil for 16 weeks. **B** Serial administrations of CCl_4_ for 8 weeks produced well developed fibrosis and early cirrhosis with deposition of mature collagen fibers (arrows). **C** At 16th week of CCl_4_ administration, the collagen fibers became thick and more prominent with well developed cirrhosis (arrows). **D** Treatment with hADSC disc for 8 weeks during CCl_4_ administration resulted in more than 50% decrease of collagen fibers and incomplete fibrous septa (arrows). The hADSC disc which is surgically implanted on right lateral lobe of the liver is visible at the bottom portion. **E** Marked reduction of cirrhosis on left lateral lobe of the liver in which the animal is treated with hADSC disc on right lateral lobe. The collagen fibers were thin with incomplete fibrous septa and partial bridging (arrows). Original magnification, × 40. **F** The staining intensity of total collagen was quantified using Image-Pro Discovery software. The data are mean ± S.D. of 10 randomly selected microscopic fields from five rats per group

Azan trichrome staining did not show any newly formed collagen deposition in the hepatic parenchyma of control rats treated with olive oil for 16 weeks (Fig. 4A). Serial administrations of CCl_4_ for 8 weeks produced well developed hepatic fibrosis and early cirrhosis with deposition of newly formed thick collagen fibers in the hepatic parenchyma (Fig. 4B). The deposition of collagen fibers became thick and more prominent with complete fibrous septa resulted in well formed cirrhosis after 16 weeks of CCl_4_ administration (Fig. 4C). Treatment with hADSC disc for 8 weeks during CCl_4_ administration completely prevented liver cirrhosis and formation of fibrous septa (Fig. 4D). Only thin collagen fibers with incomplete septa were present in rats treated with CCl_4_ and hADSC disc. The hADSC disc which is surgically implanted on right lateral lobe of the liver is visible at the bottom portion (Fig. 4D). The animals implanted with hADSC disc on right lateral lobe depicted significant decrease of liver cirrhosis on left lateral lobe also (Fig. 4E). Quantification of the staining intensity of collagen using Image-Pro Discovery software is presented as percentage square microns in Fig. 4F. The staining intensity of total collagen was significantly less (*P* < 0.001) in the animals implanted with hADSC disc as well as on left lateral lobe of hADSC group compared to animals treated with CCl_4_ for 16 weeks without hADSC disc.

### Implantation of hADSC disc remarkably reduced the expression of TGF-β1

**Fig. 5:**
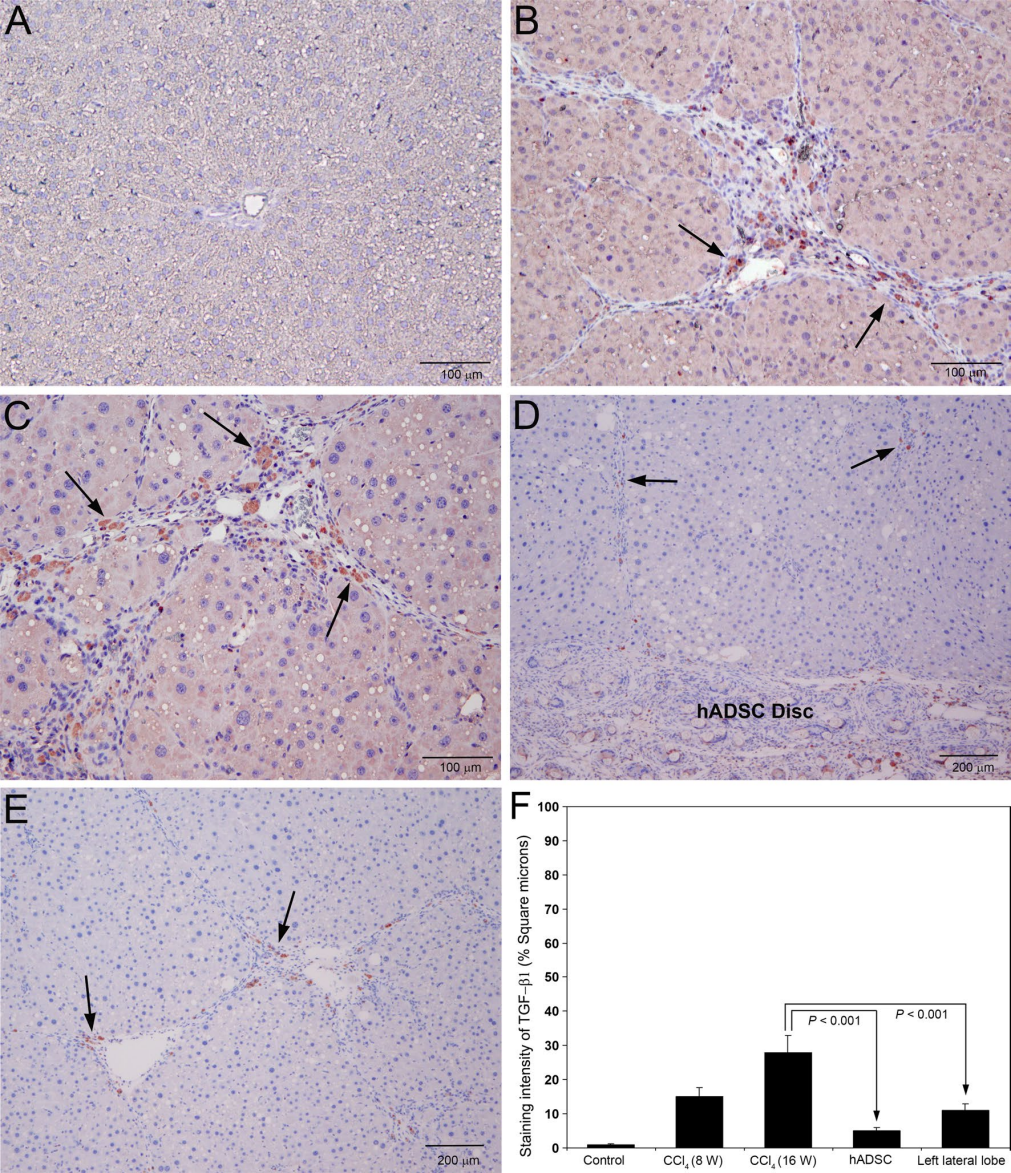
Immunohistochemical staining for transforming growth factor-β1 (TGF-β1) in rat liver sections following serial administrations of CCl_4_ and after treatment with hADSC disc. **A** Staining for TGF-β1 was completely absent in the control livers treated with olive oil for 16 weeks. **B** Marked staining for TGF-β1 was present in the fibrotic areas of rat livers treated with CCl_4_ for 8 weeks (arrows). **C** Prominent and conspicuous staining for TGF-β1 was present at 16th week of CCl_4_ administration, especially in the fibrotic zone (arrows). **D** Treatment with hADSC disc for 8 weeks showed marked reduction in staining for TGF-β1 in the hepatic parenchyma (arrows). The implanted hADSC disc is visible at the bottom portion of the image with positive staining for TGF-β1. **E** There was significant decrease in staining of TGF-β1 in the left lateral lobe, where the right lateral lobe is implanted with hADSC disc (arrows). **A**–**C** Original magnification, × 100. **D**, **E** Original magnification, × 40. **F** The staining intensity of TGF-β1 was quantified using Image-Pro Discovery software. The data are mean ± S.D. of 10 randomly selected microscopic fields from five rats per group

Transforming growth factor-β1 is considered as the most potent profibrogenic molecule inducing transdifferentiation of quiescent hepatic stellate cells into myofibroblast like cells triggering hepatic fibrosis (Xu et al. 2016). The outcome of the immunohistochemical staining of TGF-β1 during pathogenesis of CCl_4_ induced hepatic fibrosis and after the treatment with hADSC disc are depicted in Fig. 5. Staining for TGF-β1 was completely absent in the control animals treated with olive oil for 16 weeks (Fig. 5A). There was marked staining for TGF-β1 in the fibrotic zone of livers treated with CCl_4_ for 8 weeks (Fig. 5B). At 16th week of CCl_4_ administration, the staining for TGF-β1 has been remarkably increased in the fibrotic areas with intense infiltration of neutrophils (Fig. 5C). Implantation of hADSC disc for 8 weeks demonstrated marked decrease in the staining intensity of TGF-β1 in the hepatic parenchyma (Fig. 5D). A significant decrease in the staining intensity of TGF-β1 was observed in the left lateral lobe also of the livers treated with hADSC disc on the right lateral lobe (Fig. 5E). The staining intensity of TGF-β1 measured as square microns is presented in Fig. 5F. There was significant reduction (*P* < 0.001) in the staining intensity of TGF-β1 in both right and left lateral lobes of the livers treated with hADSC disc compared to the CCl_4_ administered animals without the treatment of hADSC disc.

### Application of hADSC disc inhibited activation of hepatic stellate cells

**Fig. 6:**
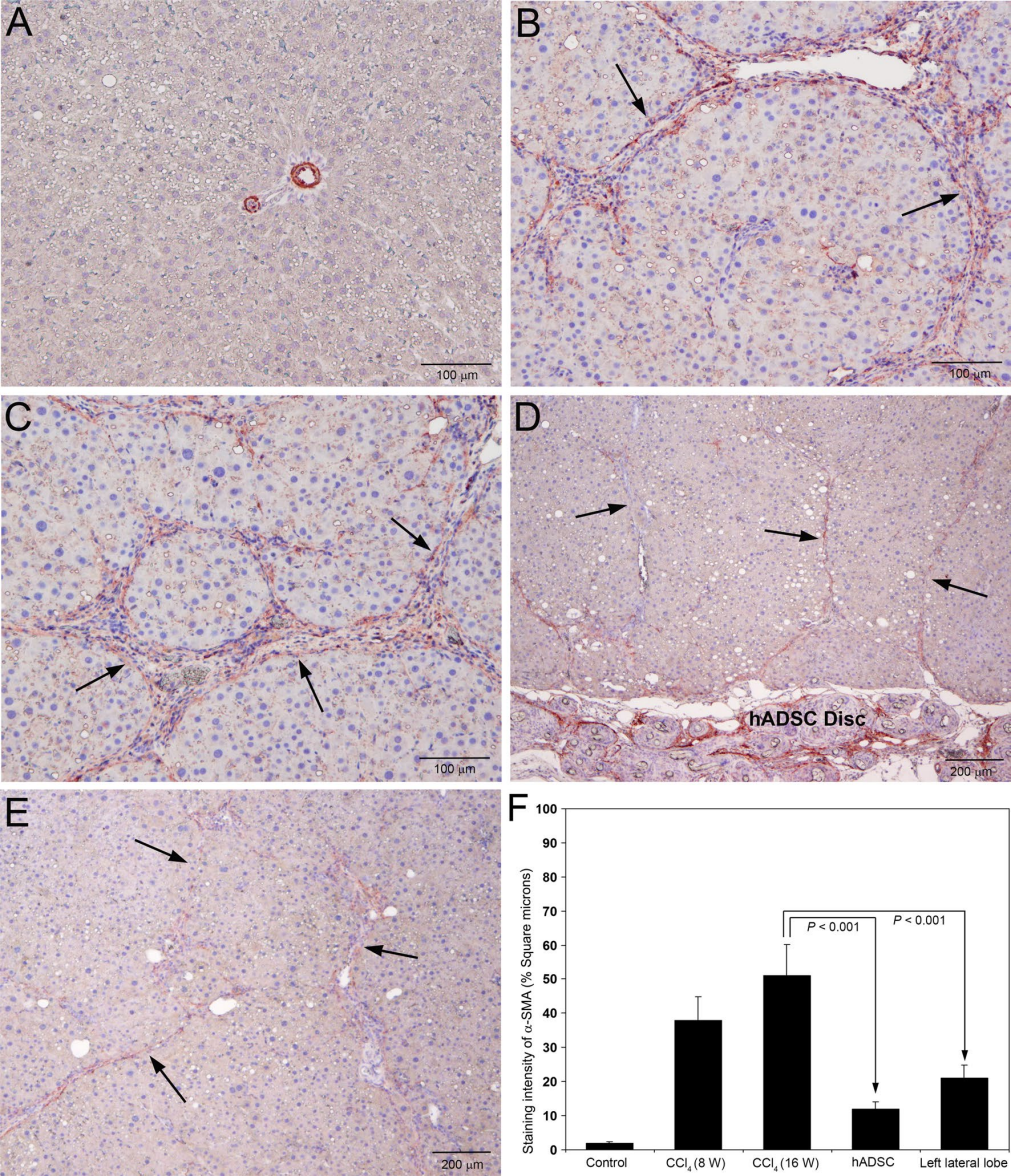
Immunohistochemical staining of α-smooth muscle actin (α-SMA) in rat liver sections following serial administrations of CCl_4_ and after treatment with hADSC disc. **A** Staining for α-SMA was completely absent in the control livers treated with olive oil for 16 weeks. **B** Remarkable staining for α-SMA was present in the fibrotic areas of rat livers treated with CCl_4_ for 8 weeks (arrows). **C** At 16th week of CCl_4_ administration, enormous and obvious staining for α-SMA was present in the periphery of thick collagen fibers (arrows). **D** Treatment with hADSC disc for 8 weeks demonstrated significant reduction in staining for α-SMA in the fibrotic zone (arrows). The implanted hADSC disc is clearly visible at the bottom portion of the image. **E** Marked decrease in staining of α-SMA in the left lateral lobe, where the right lateral lobe is implanted with hADSC disc (arrows). **A**–**C** Original magnification, × 100. **D**, **E** Original magnification, × 40. **F** Quantification of the staining intensity of α-SMA as square microns. The intensity of α-SMA staining was quantified using Image-Pro Discovery software. The data are mean ± S.D. of 10 randomly selected microscopic fields from five rats per group

The expression of α-SMA is contemplated as a marker for the activation of quiescent stellate cells associated with the initiation of hepatic fibrosis (George and Tsutsumi 2007). The results of the immunohistochemical staining for α-SMA in rat liver sections following serial administrations of CCl_4_ and after the treatment with hADSC disc are demonstrated in Fig. 6. Staining for α-SMA was totally absent in the hepatic parenchyma of control rats treated with olive oil for 16 weeks (Fig. 6A). There was remarkable and conspicuous staining of α-SMA in the fibrotic zone of rat livers treated with CCl_4_ for 8 weeks (Fig. 6B). Serial administrations of CCl_4_ for 16 weeks produced enormous and prominent staining of α-SMA, especially in the peripheral areas of fibrosis and thick collagen fibers (Fig. 6C). Implantation of hADSC disc at the 8th week of CCl_4_ administration resulted in marked reduction of α-SMA staining in the fibrotic areas (Fig. 6D). There was significant decrease in the staining intensity of α-SMA in the left lateral lobe of the liver in the animals implanted with hADSC disc on right lateral lobe (Fig. 6E). Quantification of the staining intensity of α-SMA as square microns depicted significant reduction (*P* < 0.001) in both right and left lateral lobes of the animals implanted with hADSC disc compared to the CCl_4_ administered animals without the implantation of hADSC disc (Fig. 6F).

### Implantation of hADSC disc prevented deposition of collagen type I and type III in the hepatic parenchyma

**Fig. 7:**
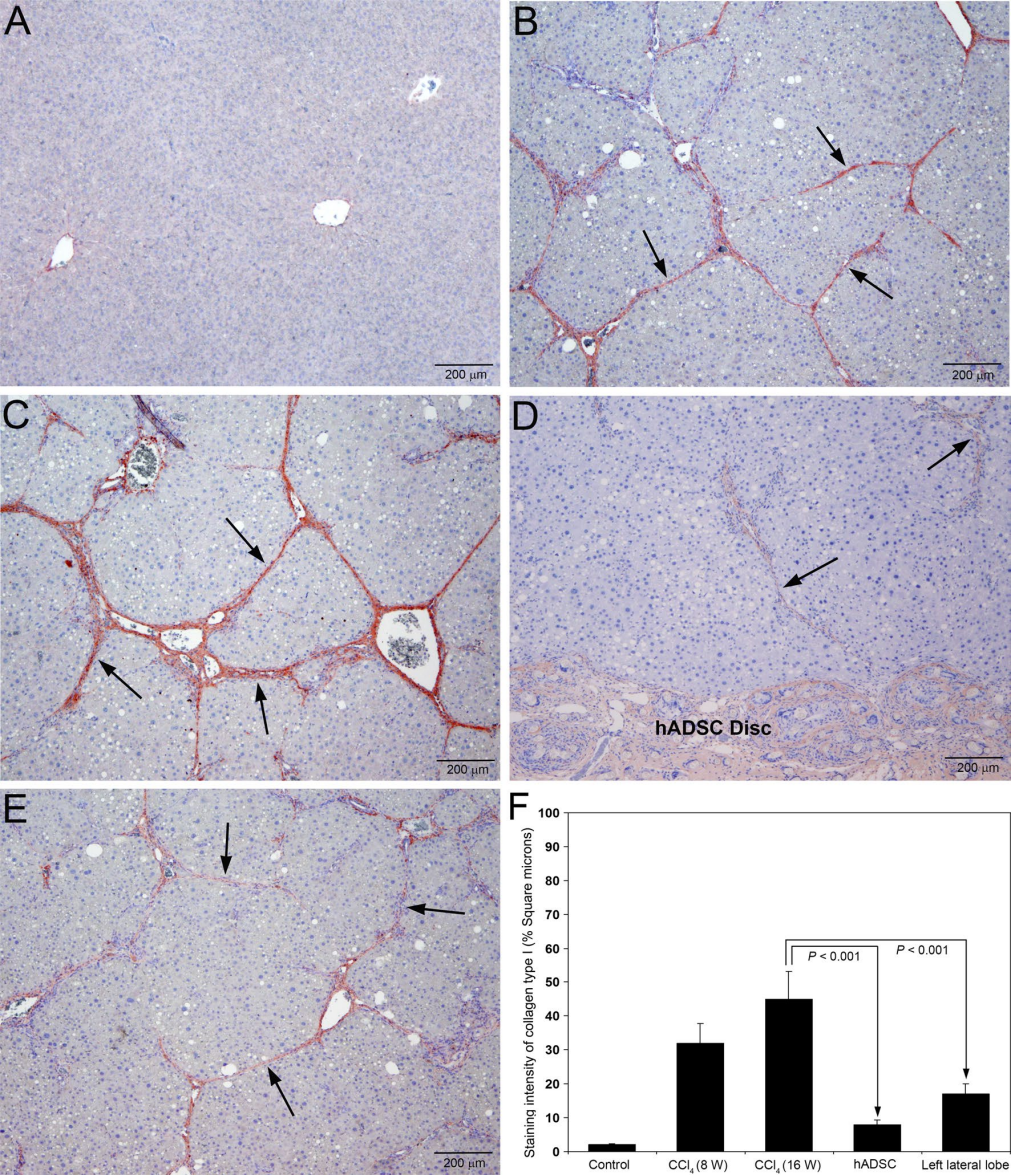
Immunohistochemical staining for collagen type I in rat liver sections following serial administrations of CCl_4_ and after treatment with hADSC disc. **A** Staining for collagen type I was absent in the hepatic parenchyma of control livers treated with olive oil for 16 weeks. **B** At 8th week of CCl_4_ administration, there was marked staining for collagen type I in the fibrotic zone indicating deposition of newly formed collagen (arrows). **C** At 16th week, the staining for collagen type I became more prominent depicting thick collagen fibers (arrows). **D** Treatment with hADSC disc for 8 weeks resulted in marked decrease of collagen type I staining indicating decreased synthesis and deposition of collagen type I (arrows). The hADSC disc with weak staining for collagen type I is visible at the bottom portion. **E** Marked reduction of staining for collagen type I in the left lateral lobe, where the right lateral lobe is implanted with hADSC disc. The collagen type I fibers were thin with incomplete fibrous septa and partial bridging (arrows). Original magnification, × 40. **F** The staining intensity of collagen type I was quantified using Image-Pro Discovery software. The data are mean ± S.D. of 10 randomly selected microscopic fields from five rats per group

**Fig. 8:**
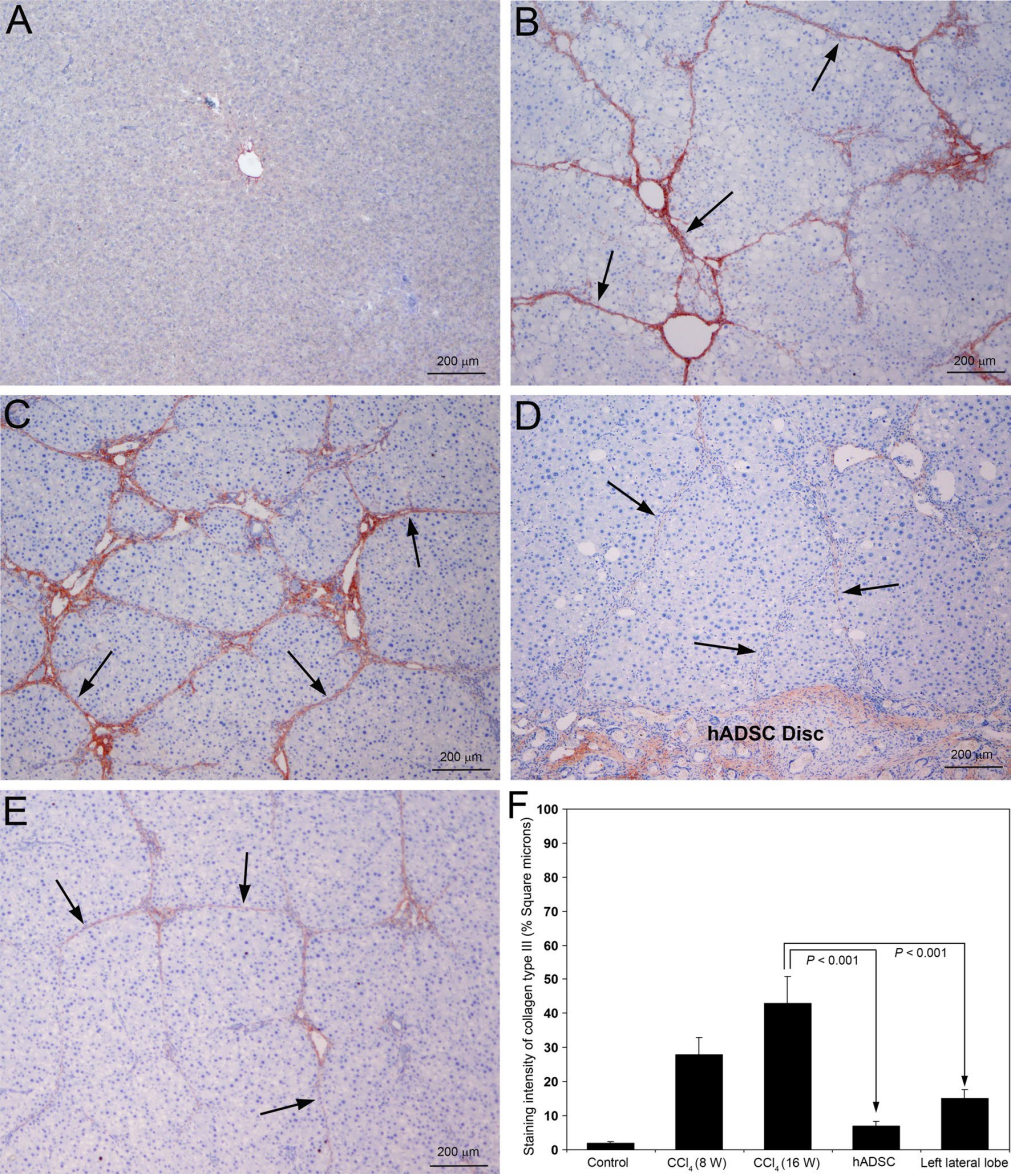
Immunohistochemical staining for collagen type III in rat liver sections following serial administrations of CCl_4_ and after treatment with hADSC disc. **A** Staining for collagen type III was completely absent in the control livers treated with olive oil for 16 weeks. **B** Prominent and marked staining for collagen type III was present in the fibrotic septa of all the rats treated with CCl_4_ for 8 weeks (arrows). **C** At 16th week of CCl_4_ administration, remarkable and conspicuous staining for collagen type III with thick fibers were present (arrows). **D** Treatment with hADSC disc for 8 weeks depicted marked reduction in staining for collagen type III with feeble and incomplete collagen fibers (arrows). **E** Significant decrease of staining for collagen type III in the left lateral lobe, where the right lateral lobe is implanted with hADSC disc. Collagen fibers were thin with incomplete fibrous septa and partial bridging (arrows). Original magnification, × 40. **F** The intensity of collagen type III staining was quantified using Image-Pro Discovery software. The data are mean ± S.D. of 10 randomly selected microscopic fields from five rats per group

The results of the immunohistochemical staining for collagen type I and type III in rats treated with CCl_4_ and after implantation of hADSC disc are presented in Figs. 7 and 8, respectively. Staining for collagen type I and type III was completely absent in the hepatic parenchyma of control rats treated with olive oil for 16 weeks (Figs. 7A and 8A, respectively).

There was prominent synthesis and deposition of both collagen type I and type III in the liver tissue at 8 weeks of CCl_4_ administration (Figs. 7B and 8B, respectively). The deposition of collagen type III was much higher compared to collagen type I as indicated in Fig. 8B as well as our earlier report on experimentally induced hepatic fibrosis (George and Chandrakasan 1996). At 16th week of CCl_4_ administration, the synthesis and deposition of both collagen types were markedly increased. However, the deposition of collagen type I was conspicuously much higher compared to collagen type III as evidenced in Fig. 7C. The collagen type I fibers were very prominent and thick compared to collagen type III fibers (Fig. 7C). Application of hADSC disc for 8 weeks during CCl_4_ administration markedly and significantly reduced synthesis and deposition of both collagen type I and type III in the hepatic parenchyma (Figs. 7D and 8D, respectively). Only thin and weak collagen fibers were present. Since the hADSC disc is made with hydroxyapatite, which is the primary component of bone that contains both collagen type I and type III, there was moderate staining for both collagen types in the implanted hADSC disc. The implantation of hADSC disc on the right lateral lobe of CCl_4_ treated animals depicted significant decrease in the staining intensity of both collagen type I and type III on the left lateral lobe also (Figs. 7E and 8E, respectively). Quantification of the staining intensity of collagen type I and type III is presented in Figs. 7F and 8F, respectively. The staining intensity of both collagen type I and type III was significantly less (*P* < 0.001) in both right and left lateral lobes of the animals implanted with hADSC disc compared to the animals without the implantation hADSC disc.

### Characterization of hADSCs at eight weeks after implantation

**Fig. 9:**
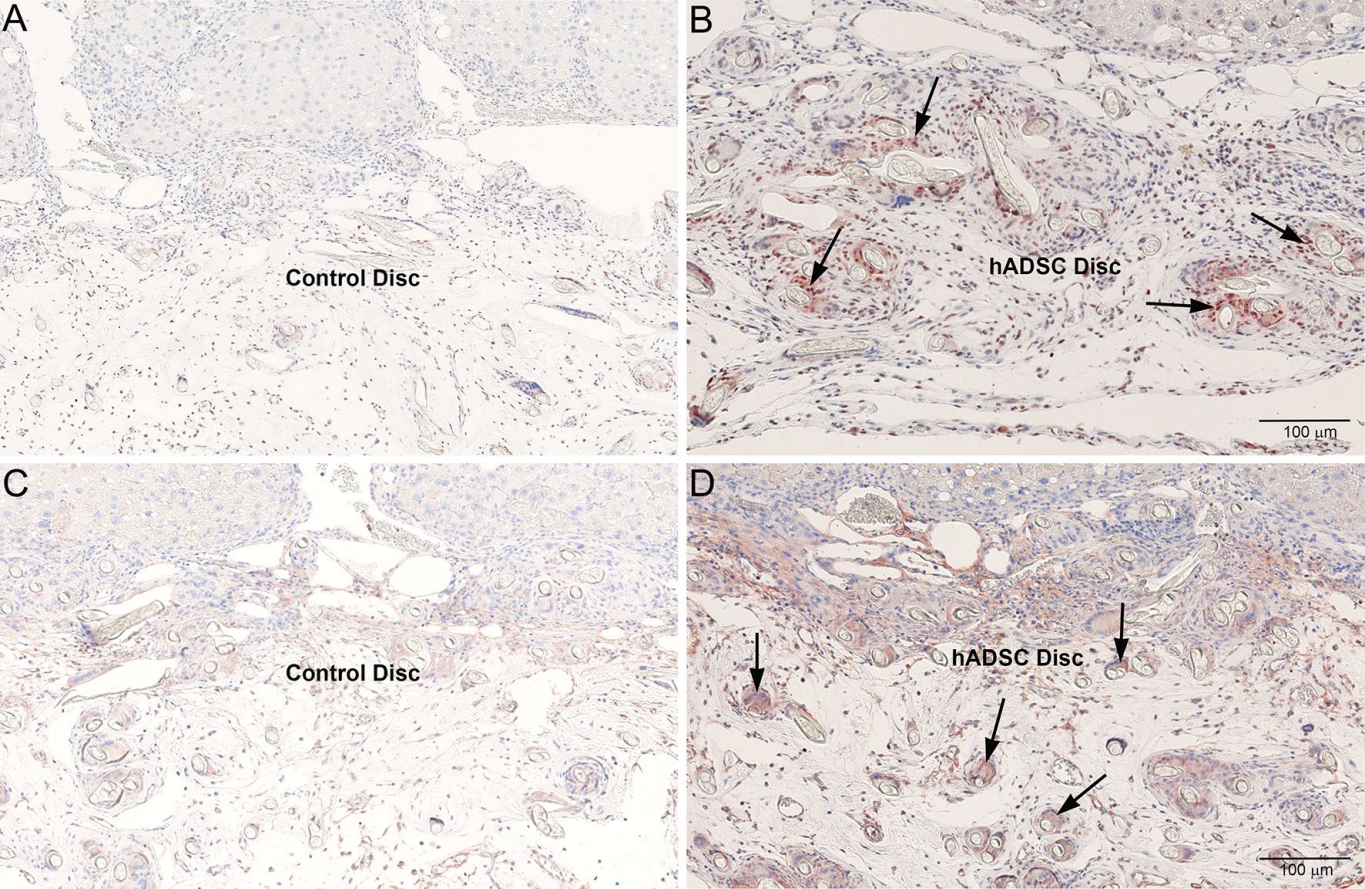
Immunohistochemical staining for human lamin B1 and CD73 at 8 weeks after implantation of hADSC disc on rat livers. **A** Implantation of disc alone without hADSCs. Staining for lamin B1 was completely absent. **B** Positive staining for human lamin B1, a characteristic highly conserved marker to demonstrate that the cells are human origin (arrows). **C** Implantation of disc alone without hADSCs. Since CD73 is expressed on subsets of T and B lymphocytes, biliary epithelial cells, and sinusoidal endothelial cells, there was focalized moderate staining for CD73 in the hepatic parenchyma. **D** Adipose-derived stem cells were stained positive for CD73 (arrows)

The results of the immunohistochemical staining for human lamin B1 and CD73 at 8 weeks after implantation of control disc without hADSCs and disc with hADSCs are presented in Fig. 9. Staining for lamin B1 was absent in the liver tissue implanted with control disc (Fig. 9A). Lamin B1, a characteristic highly conserved marker to prove human origin of the cells, was stained positively in the liver tissue implanted with hADSC disc (Fig. 9B). CD73 has been identified as a positive marker for adipose derived stem cells by the International Society for Cell Terapy (ISCT). Since CD73 is also expressed on subsets of T and B lymphocytes, biliary epithelial cells, and sinusoidal endothelial cells, there was focalized moderate staining for CD73 in the hepatic parenchyma adjacent to the implanted disc without hADSCs (Fig. 9C). Adipose stem cells were stained positive for CD73 in the liver tissue implanted with hADSC disc (Fig. 9D). The disc implanted on right lateral lobe was still adhered on the surface of the liver, which is clearly visible in Azan trichrome as well as in all immunohistochemical staining images.

## Discussion

Liver cirrhosis is a progressive disease with debilitating and life threatening outcome that affects a significant number of people worldwide. Excessive synthesis and deposition of connective tissue proteins, especially interstitial collagens in the extracellular matrix of the liver and formation of fibrous septa are hallmark of the disease (George 2008; Roehlen et al. 2020). It was reported that transplantation of mesenchymal stem cells is a novel modality for treatment of liver diseases such as inhibition of fibrosis and regulation of hepatic immune system (Yang et al. 2021). In the current study, we investigated the effects of hADSCs cultured on polyethylene disc coated with hydroxyapatite to attenuate hepatic fibrosis and early cirrhosis induced by CCl_4_ in a rat model. More than 90% of the isolated and cultured hADSCs depicted positive for CD73, CD90, and CD105 which are the major characteristic surface markers of adipose-derived stem cells (Mildmay-White and Khan 2017). In addition, there was no difference in the rate of expression of characteristic surface markers between isolated hADSCs and human ADSC procured from Lonza. Furthermore, we have demonstrated that the cultured hADSCs are capable to differentiate into adipocytes and osteocytes. These data proved that the isolated and cultured hADSCs on the implanted disc are pure multipotent stromal stem cells with regenerative and reparative functions.

Hepatic fibrosis and early cirrhosis induced by employing CCl_4_ in rats is a well established animal model to study the molecular events associated with the pathogenesis of hepatic fibrosis and cirrhosis (Dong et al. 2016). It is a highly appropriate model for screening anti-fibrotic agents and to evaluate other therapeutic approaches to arrest hepatic fibrosis and related adverse events (Raafat et al. 2015). In the present study, we employed intraperitoneal injections of 50% CCl_4_ at a dose of 0.1 ml/100 g body weight twice a week for 8 weeks to induce hepatic fibrosis and early cirrhosis in rats. As clearly demonstrated in the Azan trichrome staining as well as collagen type I and type III immunohistochemical staining, liver fibrosis and early cirrhosis was well developed at 8 weeks after the serial administrations of CCl_4_. We surgically implanted a polyethylene disc cultured with hADSCs on right lateral lobe of the experimental animals and continued the injection of CCl_4_ with the same dose once a week for another 8 weeks in order to maintain liver cirrhosis. At the end of 16 weeks, we sacrificed the animals and evaluated for attenuation of hepatic fibrosis and cirrhosis using histopathological and immunohistochemical methods. We have observed marked reduction of hepatic fibrosis not only in the lobe where hADSC loaded disc was implanted, but in the adjacent left lateral lobe also. The data suggest that application of a membranous disc loaded with hADSCs even only one lobe can decrease fibrosis in the other lobes also and the technique would be useful for the effective treatment of liver cirrhosis at early stages.

Transforming growth factor-β1 is the key signaling molecule involved in the activation and transformation of quiescent hepatic stellate cells into myofibroblast like cells and the resultant synthesis and deposition of several connective tissue proteins in the extracellular matrix of the liver (Chen et al. 2020). It was observed that TGF-β1 enhances hepatic progenitor cells during liver cirrhosis and hepatic regeneration (Yang et al. 2016). Treatment mesenchymal stem cells significantly inhibited activation of hepatic stellate cells and attenuated liver cirrhosis by inhibiting the TGF-β/Smad signaling pathway (Liu et al. 2022). In the current study, there was extensive expression of TGF-β1 especially in the fibrotic zone at 8th and 16th weeks after the serial administrations of CCl_4_ to induce hepatic fibrosis and early cirrhosis. Treatment with a disc loaded with cultured hADSCs for 8 weeks remarkably reduced the expression of TGF-β1. This data strongly indicates that certain factors released from the implanted hADSCs acted through paracrine mechanism and inhibited the expression of TGF-β1 which in turn resulted in decreased activation hepatic stellate cells subsequent attenuation of liver fibrosis and cirrhosis.

The activation and transformation of round quiescent hepatic stellate cells into myofibroblast like cells with the expression of α-smooth muscle actin filaments is considered as the first step in the pathogenesis of hepatic fibrosis (George et al. 2004). It is a dynamic process accompanied with the production of various cytokines and growth factors and marked upregulation of multiple extracellular matrix components (George 2018). The activation and trans-differentiation of quiescent hepatic stellate cells is a result of complex interplay between the hepatic parenchymal cells, dendritic cells, Kupffer cells, and extracellular matrix milieu and is a major rate limiting event in the pathogenesis and progression of hepatic fibrosis (Tsuchida and Friedman 2017). In the present study, we observed extensive and remarkable activation of hepatic stellate cells in CCl_4_ treated animals at 8th weeks and 16th weeks. Implantation of a disc loaded with cultured hADSCs markedly inhibited the activation of stellate cells. It was reported that adipose-derived mesenchymal stem cells inhibit activation and proliferation of hepatic stellate cells in vitro and ameliorate liver fibrosis in vivo (Yu et al. 2015). Various paracrine factors secreted from the multipotent hADSCs might decrease the secretion of TGF-β1 and other cytokines from hepatocytes and/or Kupffer cells that in turn reduce the activation of hepatic stellate cells and subsequent attenuation of hepatic fibrosis.

Excessive synthesis and deposition of fibrillar collagens, type I and type III in the hepatic parenchyma is the hallmark of hepatic fibrosis (George et al. 2019b, 2022). We have previously demonstrated that the deposition of type III collagen is more prominent than type I collagen in early hepatic fibrosis and fibrotic liver collagen is more cross-linked than normal liver collagen (George and Chandrakasan 1996). The development of liver cirrhosis is initiated with hepatic fibrosis, followed with bridging fibrosis, collapse of the parenchymal framework of liver, deposition of thick collagen fibers, and nodular formation. In the present study, Azan trichrome staining demonstrated deposition of newly formed thick collagen fibers in the hepatic parenchyma which is confirmed by immunohistochemical staining for collagens type I and type III. A recent report states that transplantation of adipose-derived stem cells ameliorates liver fibrosis in mice by inhibiting the expressions of α-SMA and type I collagen deposition (Yang et al. 2022). In the current study, we observed marked inhibition of the deposition of collagens type I and type III after surgical implantation of a disc loaded with cultured hADSCs on the right lateral lobe of the liver. The data strongly suggest that treatment with hADSCs during liver fibrosis could prevent progression to liver cirrhosis and the related adverse events such as development of hepatocellular carcinoma.

In the present study, we demonstrated through several images that the surgically implanted disc loaded with cultured hADSCs was still adhered on the surface of the liver even after 8 weeks of treatment. In order to confirm whether cells on the disc are still hADSCs or differentiated to hepatic progenitor cells or hepatocytes, we stained the cells with anti-human lamin B1 and CD73 antibodies employing immunohistochemical techniques. Lamin B1 is a highly conserved characteristic marker to prove human origin of the cells and CD73 is a distinctive marker to identify adipose-derived stem cells. The strong positive staining for lamin B1 and CD73 indicated that the characteristics of hADSCs are maintained even after 8 weeks of implantation. In the current study, a non-biodegradable polyethylene-polypropylene disc coated with hydroxyapatite was used in order to retain the structure of the disc to hold the cells during culture and also while implantation. A biologically derived and biodegradable honeycomb collagen scaffold employing in tissue engineering and cell based therapies (George et al. 2008; Itoh et al. 2001) would be ideal for differentiation of hADSCs into hepatic progenitor cells or hepatocytes after implantation. A biodegradable scaffold that does not elicit any immunological reactions can eventually disappear completely after implantation and the differentiated cells could integrate with the host tissue (Kakudo et al. 2008).

The preclinical and clinical research data of MSCs in the treatment of liver fibrosis and cirrhosis indicate that administration of MSCs is a promising therapeutic approach to inhibit the progression of hepatic fibrosis and cirrhosis. However, the exact mechanism of how MSCs attenuates hepatic fibrosis and cirrhosis is not clear. Several studies have reported that the antifibrotic effects of MSCs therapy may be attributed to various types of trophic factors and cytokines produced by the stem cells (Tolar et al. 2010; Hu et al. 2019). It was observed that hepatocyte growth factor over-expressed MSCs has increased therapeutic effect against experimentally induced hepatic fibrosis compared to normal MSCs (Kim et al. 2014). MSCs reverse liver fibrosis and improve liver function mainly through differentiation into hepatocytes, immune regulation, secretion of cytokines, and growth factors, reduction of hepatocyte apoptosis, and promotion of hepatocyte regeneration (Zhu et al. 2021). In a recent review, it was stated that cell-free therapies based on extracellular vesicles (exosomes) derived from MSCs could be efficiently employed for the treatment of cirrhosis and liver failure rather than cell based therapies (Watanabe et al. 2021). Exosomes could be safely administered in large doses and are able to infiltrate target organs. We have observed that exosome-mediated delivery of anticancer agents effectively induced cell death and regressed intrahepatic tumors in experimental animal model (George et al. 2018). In the current study, even though we implanted the disc with cultured hADSCs only on the right lateral lobe of the liver, we observed significant decrease of fibrosis and early cirrhosis in the left lateral lobe also. This could be due to the diffusion of cytokines and growth factors produced by the hADSCs to the other lobes of the liver.

## Conclusions

The results of the present study demonstrated that implantation of a disc loaded with cultured hADSCs remarkably reduced hepatic fibrosis and early cirrhosis and improved liver functions. Multiple factors such as production of several cytokines, growth factors, antioxidants, as well as immune regulation from hADSCs could be responsible for the arrest of fibrosis and regeneration of hepatocytes. Furthermore, various pleiotropic and paracrine factors secreted from the multipotent hADSCs seem to serve as reparative functions in the attenuation of liver cirrhosis. The data demonstrated that treatment with hADSCs can be successfully used as a potent therapeutic method to arrest liver cirrhosis and related adverse events.

## Data Availability

The data will be available for verification upon request.

## Abbreviations

AEC: 3-Amino-9-ethylcarbazole
ALT: Alanine aminotransferase
APC: Allophycocyanin
AST: Aspartate aminotransferase
CCl_4_: Carbon tetrachloride
CD: Cluster of differentiation
DAB: 3,3′-Diaminobenzidine
EDTA: Ethylene diamine tetraacetic acid
FACS: Fluorescence-activated cell sorting
FITC: Fluorescein isothiocyanate
hADSCs: Human adipose derived stem cells
HDFC: Human dermal fibroblast cells
H&E: Hematoxylin and eosin
HLA: Human-leukocyte antigen
hMSC: Human mesenchymal stem cell
4-HNE: 4-Hydroxy-2-nonenal
mAbs: Monoclonal antibodies
MDA: Malondialdehyde
MSCs: Mesenchymal stem cells
PE: Phycoerythrin
PE-PP: Polyethylene-polypropylene
TGF-β1: Transforming growth factor-β1
α-SMA: α-Smooth muscle actin
